# Dyspnea due to an uncommon vascular tumor: leiomyosarcoma of the infrahepatic vena cava inferior

**DOI:** 10.1186/s40792-020-00896-9

**Published:** 2020-06-16

**Authors:** Samra Gafarli, Dorian Igna, Mathias Wagner, Adriana Nistor, Matthias Glanemann, Barbara Stange

**Affiliations:** 1 Department of General-, Visceral-, Vascular- and Pediatric Surgery, Saarland University, Kirrberger Straße 100, D-66421 Homburg, Saar Germany; 2 Department of Pathology, Saarland University, Homburg, Saar Germany

**Keywords:** Dyspnea, Leiomyosarcoma, Inferior vena cava, Surgery

## Abstract

**Background:**

Leiomyosarcoma (LMS) of the inferior vena cava (IVC) is a rare malignancy that originated from the smooth muscle tissue of the vascular wall. Diagnoses, as well as, treatment of the disease are still challenging and to date, a radical surgical resection of the tumor is the only curative approach.

**Case report:**

We report on the case of a 49-year old male patient who presented with suddenly experienced dyspnea. Besides bilateral pulmonary arterial embolism, a lesion close to the head of the pancreas was found using CT scan, infiltrating the infrahepatic IVC. Percutaneous ultrasound-guided biopsy revealed a low-grade LMS. Intraoperatively, a tumor of the IVC was observed without infiltration of surrounding organs or distant metastases. Consequently, the tumor was removed successfully, by en-bloc resection including prosthetic graft placement of the IVC. Histological workup revealed a completely resected (R0) moderately differentiated LMS of the IVC.

**Conclusion:**

LMS of the infrahepatic IVC is an uncommon tumor, which may present with dyspnea as its first clinical sign. Patients benefit from radical tumor resection. However, due to the poor prognosis of vascular LMS, a careful follow-up is mandatory.

## Introduction

Sarcomas are connective tissue origin tumors representing only 1% of all adult malignancies [1]. Leiomyosarcoma (LMS) is a subtype of soft tissue sarcomas (STS) which accounts for less than 8–20% of all STS and mostly affects the gastrointestinal tract, uterus, skin as well as blood vessels [2, 3].

Primary LMS of the inferior vena cava (IVC) is a rare type of vascular LMS with only a few hundred cases in the literature since its first description in 1871 by Perl as an autopsy finding [4, 5]. Initially, these neoplasms were considered inoperable; however, improvements in surgical techniques and perioperative management allowed to the first surgical tumor resection which was carried out in 1928 by Melchior [6].

The diagnosis is mostly challenging because of untypical clinical symptoms [4]. In addition, the use of modern imaging methods is also complex and only histopathological examination can clearly identify this malignancy [7, 8]. Radical surgical approach predominates as the only curative therapy option by complete resection with a safety margin; however, still with a poor prognosis with median overall survival of around 23–71 months [4, 9–11].

### Case report

A 49-year-old male patient presented with suddenly experienced dyspnea in the internal medicine emergency unit. His medical history included a stage IIA nodular sclerosing type of classical Hodgkin lymphoma (NSCHL), splenectomy at the age of 9, implantation of a cardiac resynchronization therapy pacemaker (CRT-P) at the age of 35, a stage IA/E diffuse large B-cell lymphoma (DLBCL) type of non-Hodgkin lymphoma (NHL) near the left maxillary tuber at the age of 37, and bioprosthetic aortic valve replacement at the age of 44. Transthoracic echocardiogram showed a normal ejection fraction of 60% with normal positioned CRT-P with good function. Furthermore, laboratory analysis revealed D-Dimer 6.32 mg/l. Deep vein thrombosis was excluded by the duplex sonography examination.

**Fig. 1:**
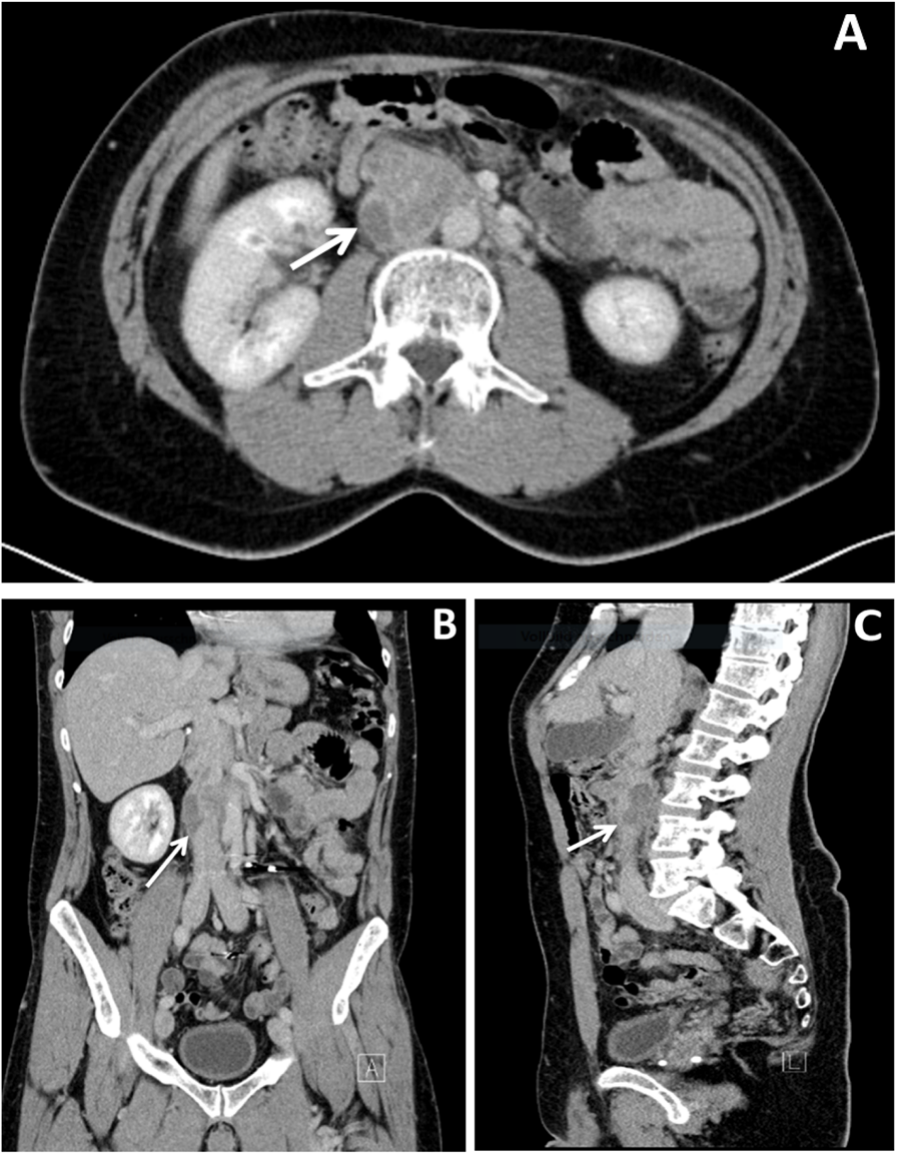
CT scan demonstrating a lesion (41.7 × 30.1 × 57.8 mm) with IVC infiltration and thrombus within the IVC (white arrows). **a** Axial. **b** Coronal. **c** Sagittal

**Fig. 2:**
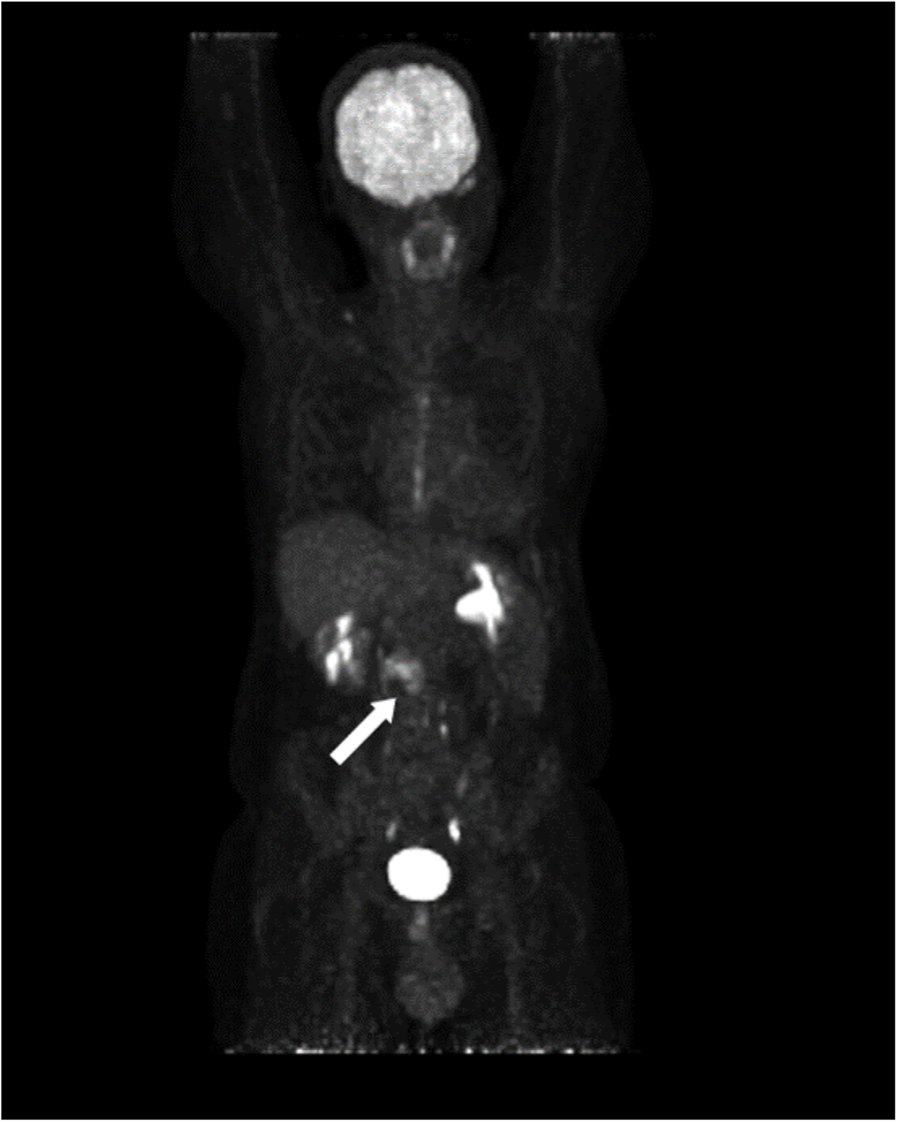
PET-CT scan demonstrating an infrarenal located, glucose hypermetabolic mass without evidence of distant metastases (white arrow)

Subsequent computed tomography (CT) scan revealed bilateral pulmonary arterial embolism in lower lobes. Moreover, a solid tumorous mass close to the pancreas head was observed, along with infiltration into and a significant portion of thrombus material inside of the IVC (Fig. 1). A positron emission tomography–computed tomography (PET-CT) scan revealed a glucose hypermetabolic mass without any evidence of distant metastases (Fig. 2). Endoscopic ultrasound (EUS) revealed an inhomogeneous, hypoechoic tumor (42 × 30 mm) with infiltration of the IVC without abnormal findings in the head of the pancreas. A EUS-guided fine needle biopsy was not feasible due to crossing blood vessels. For this reason, a percutaneous ultrasound-guided biopsy was done and a low-grade LMS was diagnosed histologically.

**Fig. 3:**
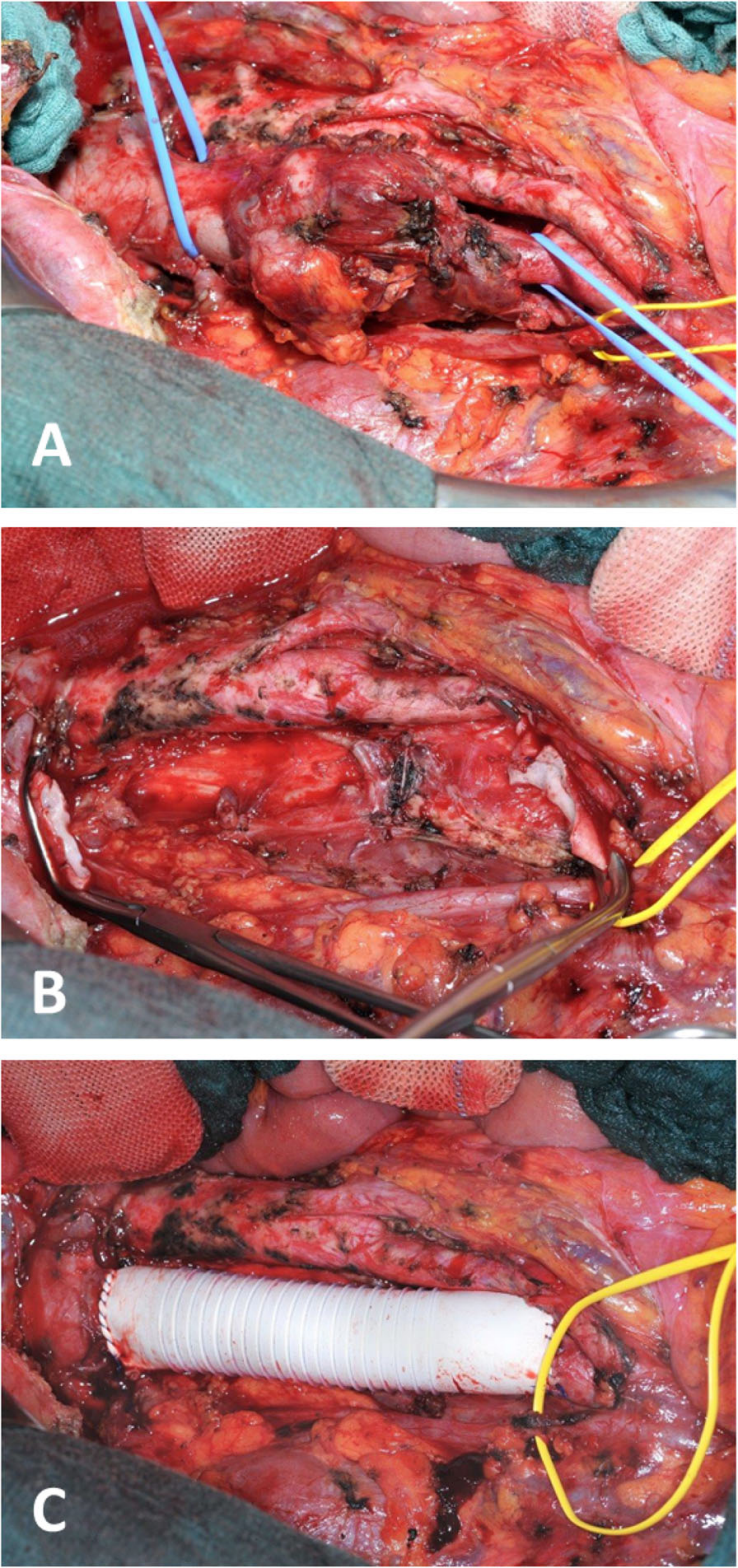
Intraoperative situs. **a** Presentation of the IVC after Cattell maneuver (blue vessel loops on the proximal side infrarenal and on the distal side prebifurcation, yellow vessel loop for isolation of the right ureter). **b** Situs after en-bloc tumor resection between Satinsky vena clamps. **c** IVC-reconstruction using a 20-mm PTFE vascular graft

After extensive diagnosis, the case was presented in the interdisciplinary tumor board. By taking into account the patient’s general condition, a decision on surgical treatment was made. Using midline laparotomy, the tumor region was exposed after Cattell maneuver (Fig. 3a). Hereby, the tumor mass was identified in the retroperitoneal space, being infrarenal part of the IVC (segment I), whereas the duodenum and head of the pancreas were separated from the tumor without problems. Then, en-bloc resection of the tumor was performed using two Satinsky vena clamps (Fig. 3b). Prosthetic replacement of the IVC was performed using a 20-mm diameter ring-enforced polytetrafluoroethylene (PTFE) vascular graft (Fig. 3c).

**Fig. 4:**
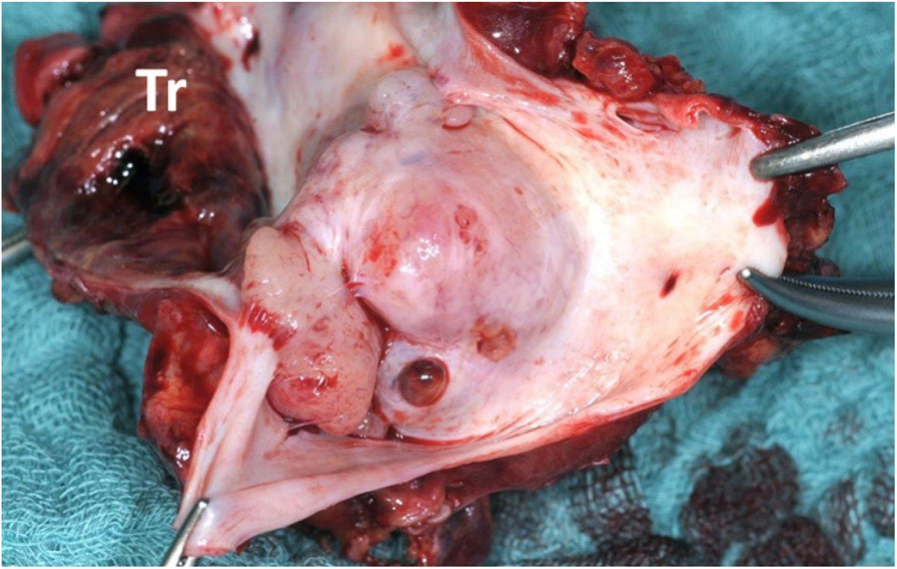
Intraluminal part of the mass with thrombotic material (Tr) adherent to the cranial end of the lesion

**Fig. 5:**
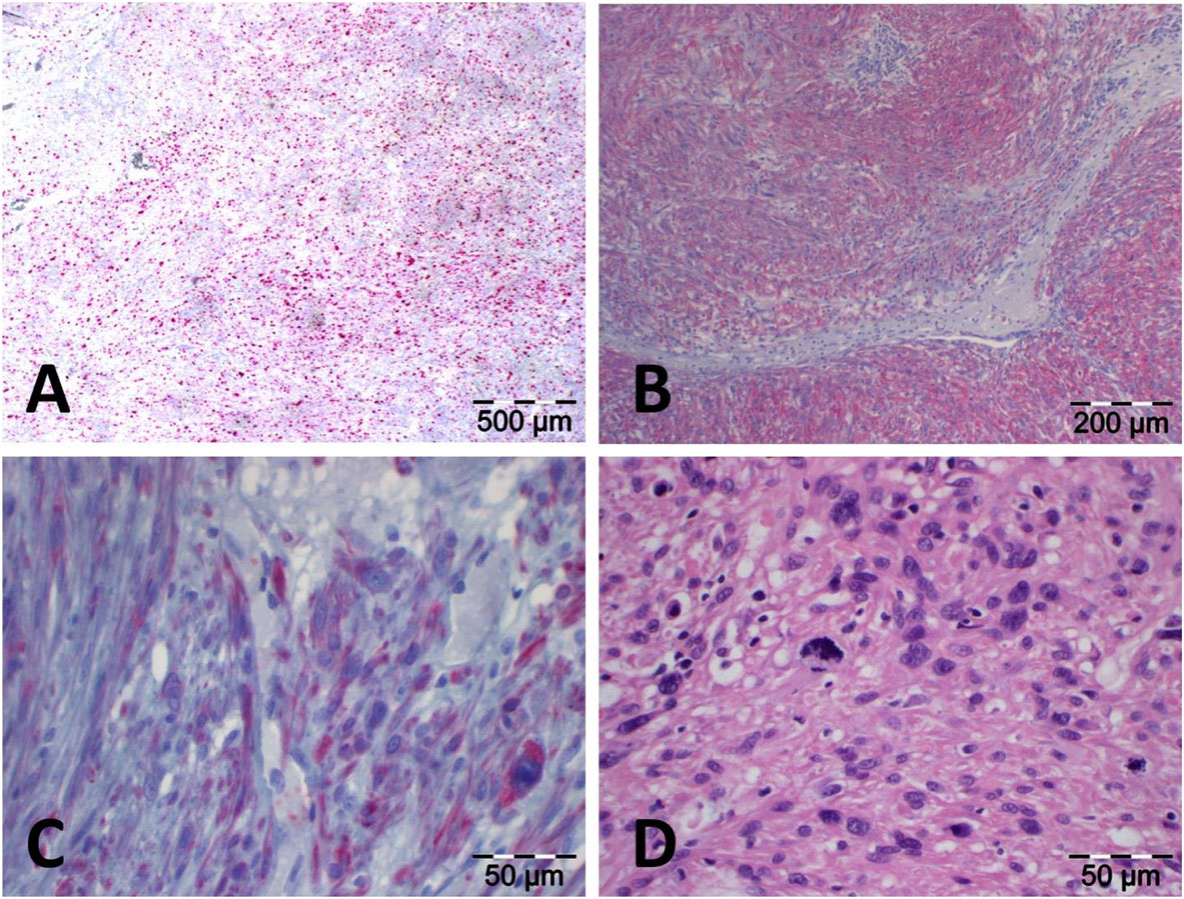
Histopathological findings. All staining was carried out on formalin-fixed paraffin-embedded (FFPE) tissue. **a** Monoclonal antibodies to the Ki67 antigen (Anti-Ki67) were applied to flag cells in the G1, S, G2, and M phase of their cell cycle, but not in the resting phase G0. Despite the fact that intraobserver and interobserver reproducibility of the visual assessment of Ki67 expression in IHC are low [12], there was a consensus that the tumor showed considerable activity. **b** The tumor cells revealed a specific, strong, and diffuse cytoplasmatic reactivity for antibodies to smooth muscle actin (Anti-α-Actin) as seen in cases of LMS. **c** The cytoplasmatic reactivity of tumor cells with antibodies to desmin (Anti-Desmin) as shown here might be associated with improved survival as the expression of this antigen can help predict the outcome by segregating moderately differentiated LMS into two groups, those that behave like well-differentiated tumors by expressing desmin and those that behave like poorly differentiated tumors by not expressing it [13]. **d** Hematoxylin and Eosin (H&E) staining revealed tumor cells with pleomorphic nuclei and eosinophilic cytoplasmata

Thrombotic material, the putative source of pulmonary artery embolism, was found adherent to the cranial margin of an intraluminal mass (Fig. 4). The IVC wall contained a 4.2 × 3.8 × 3.3 cm predominantly intraluminal mesenchymal tumor (Fig. 5). Layers of connective tissue and endothelium clearly separated the tumor cells from the bloodstream. Despite the history of NSCHL and DLBCL, no traces of Epstein-Barr Virus (EBV) could be detected in the lesion, ruling out an EBV-associated smooth muscle tumor (EBV SMT) [14–18]. The tumor was hence classified as a moderately differentiated LMS of the IVC. The postoperative course was uneventful and the patient was discharged at postoperative day 9.

A follow-up CT scan 8 months after surgery, however, revealed evidence of multiple, bilobar, intense glucose hypermetabolic liver metastases but no relapse at the primary tumor site. For this reason, the patient underwent chemotherapy with gemcitabine/docetaxel (two cycles) and hereafter with gemcitabine as monotherapy in terms of an individual treatment approach. No pulmonary filiae have been found so far, supporting the notion that tumor cells might not have contributed to the clinical course of the aforementioned bilateral pulmonary arterial embolism. Thereby, the patient is alive 20 months after surgery, with a stable disease of the liver metastases without any other distant metastases and local tumor recurrence.

## Discussion

LMS of the IVC is a rare malignant tumor. Due to the slow growth and mutable anatomic appearance, this malignancy clinically presents with ambiguous signs or is just asymptomatic [19]. Not surprisingly, approximately 10% of all cases are diagnosed incidentally [4]. In the same line of evidence and therefore highly remarkable, the first clinical sign of the tumor disease in our patient was dyspnea due to recurrent episodes of embolism, and certain diagnostic tools were required to identify the causative source of embolism.

Once diagnosed, these neoplasms were mostly regarded as inoperable tumors. Since the first surgical tumor resection by Melchior in 1928, however, and due to several improvements in surgery, anesthesiology and intensive care medicine, complete tumor resection with tumor-free margin is considered the primary and only curative treatment approach nowadays [6, 20].

With respect to the literature, data on this tumor entity is rare with less than 1.000 published cases. Interestingly, only a few reports comprise a higher number of patients. In 1991, Mingoli et al*.* were the first to publish a series of 144 patients suffering from LMS of the IVC [21, 22]. Most patients were female (about 2/3) with a median age of 55 years. The clinical symptoms were unspecific or almost lacking. Due to its rarity in consequence, they recommended the establishment of an international registry to study the pathogenesis and the natural history of this disease. In 1997, they again reported on 218 patients suffering from LMS of the IVC and who underwent radical surgical resection [21, 22]. Two decades later in 2015, Wachtel et al. reported on almost 377 cases [4].

Both reported that the majority of patients complained of unspecific abdominal pain (up to 60%) besides asymptomatic features [4]. The respiratory complaint was described by Wachtel et al. as a non-specific clinical symptom in 5.1% of the cases [4]. Not surprisingly in this context and typically for the heterogeneity of clinical presentation is the fact that LMS may even present with dyspnea as first clinical sign due to recurrent episodes of embolism as a consequence of intraluminal tumor growth and formation of local thrombosis, as occurred in our patient [8, 23–25].

Despite the fact that EBV may be associated with various malignancies [17, 18, 26–28], combinations of Hodgkin and/or non-Hodgkin lymphoma with LMS may occur without the virus. EBV negativity in our patient suggests that the clinical course was not modified by the virus.

Meaningful seems to be the site of IVC affection by the tumor in terms of patient survival. In this context, three groups of IVC segments were classified according to tumor localization: segment I—infrarenal IVC, segment II—inter- and suprarenal IVC, segment III—suprahepatic IVC and cardiac involvement [22, 29]. Patients with segment II tumor localization experienced the highest 5-year survival rates (56.7%) compared with patients with segment I tumor localization (37.8%), as reported by Mingoli et al. [30]. Additionally, segment III tumors were mostly inoperable and the median survival was only one month in this group of patients [30]. The high survival in the segment II group might be explained by the assumption that a tumor which is located near to abdominal organs and structures might earlier experience clinical symptoms, e.g., by tumor compression, resulting in early diagnosis and therapy. Remarkably, both Wachtel et al. and Mingoli et al. had described no survival benefit from radiation and chemotherapy treatment [4, 31]. Although there is no evidence showing a long-term survival improvement from adjuvant therapy, it can be considered an individual treatment approach to reduce or stabilize the recurrence of the disease [32, 33].

## Conclusion

LMS of the IVC is a rare disease and still challenging, not only to diagnose but also to cure. It is characterized by a variety of different, unspecific clinical signs. Once diagnosed, these patients should be discussed in a multidisciplinary tumor board to evaluate tumor resectability. In spite of the absence of clinical trials, the actual data support surgical resection of the tumor as a unique option for treatment. Therefore, complete tumor resection is considered the only curative therapy approach, since chemotherapy and/or radiotherapy by themselves tend to show no better survival rates [5, 11, 31, 34–36]. Indeed, radical surgical treatment can be beneficial, provided a precise patient selection and an experienced, interdisciplinary team.

## Data Availability

Not applicable.

## Abbreviations

CD: Cluster of differentiation
CRT-P: Cardiac resynchronization therapy pacemaker
CT: Computed tomography
DLBCL: Diffuse large B-cell lymphoma
EBV: Epstein-Barr virus (syn.: type 4 human herpesvirus, HHV-4)
EUS: Endoscopic ultrasound
FFPE: Formalin-fixed paraffin-embedded
FNCLCC: French Federation of Comprehensive Cancer Centers
IHC: Immunohistochemistry
ISH: In situ hybridization
IVC: Inferior vena cava
LMP: Late membrane protein of EBV
LMS: Leiomyosarcoma
NHL: Non-Hodgkin lymphoma
NSCHL: Nodular sclerosing type of classical Hodgkin lymphoma
PET-CT: Positron emission tomography–computed tomography
PTFE: Polytetrafluoroethylene
TNM: A classication system to describe size and spread of cancer
pTNM: TNM classification based on histopathologic examination
STS: Soft tissue sarcomas
UICC: Union Internationale Contre le Cancer
